# A Syndemic Clustering of Adversities on Suicide Risk among YMSM Living with HIV in Bangkok: A Causal Latent Class Analysis

**DOI:** 10.1007/s10461-024-04516-7

**Published:** 2025-01-09

**Authors:** Doug H. Cheung, Worawalan Waratworawan, Yamol Kongjareon, Kai J. Jonas, Sin How Lim, Alexis N. Reeves, Thomas E. Guadamuz

**Affiliations:** 1 Jockey Cub School of Public Health, Chinese Universirty of Hong Kong, Hong Kong SAR, China; 2 Mahidol Center for Health, Behavior and Society, Faculty of Tropical Medicine, Mahidol University, Bangkok, Thailand; 3 Department of Society and Health, Faculty of Social Sciences and Humanities, Mahidol University, Nakhon Pathom, Thailand; 4 Department of Work and Social Psychology, Maastricht University, Maastricht, The Netherlands; 5 Department of Social and Preventive Medicine, Universiti Malaya, Kuala Lumpur, Malaysia; 6 Department of Epidemiology and Population Health, Stanford University School of Medicine, Palo Alto, CA USA; 7 John F. Kennedy School of Government, Harvard University, Cambridge, MA USA

**Keywords:** HIV/AIDS, Men who have sex with men, Suicide prevention, Thailand, Causal Inference

## Abstract

This study investigated the clustering of psychosocial adversities and their synergistic effect with depression on suicidality in a 12-month prospective cohort (*N* = 214) of YMSM living with HIV in Bangkok, Thailand. Latent class analysis identified subgroups with distinct combinations of adversities, including bullying, intimate partner violence, substance use, HIV stigma, low social support, histories of sex work, and below-income status. Significant interactive synergism were found as hypothesized, supporting a syndemic effect with qualitatively increasing levels of adversities on suicidality (score range: 3–17) over the 12 months. The interaction between moderate adversity clustering and depression (β_ow_ = 2.50, 95% CI: 1.12–3.88) and high adversity clustering and depression (β_ow_ = 3.61, 95% CI: 1.12–6.09) indicated that the impact of depression on suicidality was modified by pre-existing adversities. The findings suggest that, while a multi-component intervention addressing psychosocial problems is ideal, effective depression treatment alone could significantly reduce suicidality among YMSM living with HIV.

**Supplementary Information:**

The online version contains supplementary material available at 10.1007/s10461-024-04516-7.

## Resumen

Este estudio investigó el agrupamiento de adversidades psicosociales y su efecto sinérgico con la depresión en la suicidabilidad en una cohorte de hombres jóvenes que tienen relaciones sexuales con hombres (HSH) viviendo con VIH en Bangkok, Tailandia (*N* = 214). Se identificaron subgrupos que presentan combinaciones de adversidades como acoso, violencia de pareja, consumo de sustancias, estigma del VIH, bajo apoyo social, historia de trabajo sexual y bajos ingresos. Tres modelos estructurales marginales estimaron los efectos conjuntos del agrupamiento de adversidades y la depresión en la suicidabilidad durante 12 meses. Se encontraron efectos sinérgicos significativos, evidenciando un efecto sindémico en la suicidabilidad: interacción entre adversidades moderadas y depresión (β_ow_: 2.50; IC 95%: 1.12–3.88) y entre adversidades altas y depresión (β_ow_: 3.61; IC 95%: 1.12–6.09). Los hallazgos sugieren que tratar la depresión podría mejorar significativamente la suicidabilidad en este grupo vulnerable.

## Introduction

Men who have sex with men (MSM) are at disproportionately high risk of suicide. A systematic review found that lesbian, gay, and bisexual people have a two-fold higher risk of suicide attempts than their heterosexual counterparts [1]. The lifetime prevalence of suicidal ideation among MSM is 19.1% [2], which is 2-7.5 times higher than [3]. The heightened risk of suicide among MSM can be explained by the complex and intertwined clustering of psychosocial adversities faced by MSM attributable to their sexual minority status [4, 5]. The syndemic theory proposed by Singer conceptualizes the interconnectedness and mutually enforcing relationships of Substance Abuse, Violence and HIV/AIDS-related risk behaviors (termed the SAVA syndemic) working in tandem to exacerbate health among the urban poor in the US [6, 7]. Syndemic theory has been widely utilized to explain health disparities across and within marginalized populations, particularly among MSM in the US [8].

The syndemic approach has also been used to predict and explain the risk of suicide among MSM and other minority populations [4, 9–13]. Mustanski et al. have shown that depressive symptoms mediate the effects of bullying and victimization to increase the risk of suicide attempts among MSM youth. Specifically, when bullying and victimization, depressive symptoms, substance use, and other SAVA adversity indicators were modelled as a “syndemic latent factor,” it more reliably predicted risks of suicide attempts among MSM than heterosexual youth in the US [4, 14]. However, Ferlatte et al. found that when summing the counts of various health challenges (smoking, party drug use, depression, anxiety, STIs, condomless anal sex, and living with HIV) reported by MSM, they were dose-dependently associated with suicidal ideation and suicide attempts, thus showing an accumulation of suicide risk by increasing the syndemic burden [9]. Although this sum score approach is widely used, it has been challenged because of its statistical assumptions [15], inadequate representation of key concepts of syndemic theory, and contentious implications in informing effective public health interventions and policies [16–18].

A central tenet of syndemic theory emphasizes the joint causal effects that can arise from the synergistic interactions between biological, social, and psychological conditions in explaining health disparities across marginalized populations, such as the distribution of HIV/AIDS as socially patterned and contextually linked to poverty and other structural determinants of health [8, 18]. In this conceptualization, an ideal intervention would aim to address all syndemic conditions through a multi-component approach [16]. However, addressing a single health risk or exposure in isolation may still provide benefits, even without intervening in other interacting factors, a relevant consideration for resource-limited settings where a multi-component approach to interventions may not be feasible or suited to specific subpopulations, because it requires a long development timeline. In Thailand, a demonstration study integrated mental health treatment into existing HIV care services for 31 HIV-positive adolescents (12% identified as LGBT) who screened positive for mental health disorders [19]. One year after referral to psychiatric care, 42% no longer showed significant psychiatric symptoms, whereas 26% showed improved mental health. Scaling up such integrated mental health-HIV care could potentially benefit young Thai men who have sex with men (YMSM) living with HIV. However, the unique and clustered psychosocial adversities and social contexts experienced by this group present challenges to the effectiveness of mental health approaches designed for the general population.

In addition to the SAVA syndemic conceptualization, minority stress theory, as proposed by Brooke and later propagated by Meyer, posits that depression and internalized stigma act as proximal stressors that mediate and/or interact with more distal minority stress processes, such as experienced victimization and discrimination [20, 21]. Studies among MSM living with HIV have found a heightened suicide risk associated with higher levels of reported HIV-related stigma [22–25]. Furthermore, research on LGBT adolescents in Thailand has found that loneliness and poor relationship quality tend to cluster with stress and victimization [26, 27]. This is consistent with the interpersonal-psychological theory of suicide, which posits that diminished interpersonal well-being mediates the relationship between depression and suicide risk among LGB youth in the US [28]. Our own research with YMSM living with HIV in Bangkok also found that higher levels of perceived social support cross-sectionally moderated associations between depression, HIV stigma, and ART adherence [29]. Therefore, clustering syndemic, such as SAVA, further promotes the clustering of adversities proceeding with YMSM’s HIV status by diminishing their levels of social support, together with internalized stigma. This then synergistically interacts with proximal stress processes such as depression, potentially elevating the risk of suicide in this population.

Another core tenet of syndemic theory emphasizes the importance of understanding local population-specific contextual factors. This approach helps understand why certain adversities cluster together in the first place, influencing the likelihood of adverse interactions occurring within places, times, and among the most affected subgroups [18, 30]. Beneath Bangkok’s veneer as a ‘gay paradise,’ more implicit forms of social marginalization, such as homonegativity, persist in places such as schools, workplaces, and homes [31, 32]. The majority of LGBT individuals reported experiencing discrimination in educational and employment settings that diminished their economic well-being and increased reliance on potentially unaccepting family members, a process that likely aggregates various forms of stress and interpersonal conflict [31, 33–35]. Consequently, some individuals may resort to sex to gain financial independence. Although Thailand provides 12 years of free basic education, professions such as medicine, law enforcement, and other white-collar work were identified as among the least accessible to LGBT youth, while careers in beauty salons, massage parlors, and sex work were seen as the most viable options [31]. While sex work has been postulated as a “syndemic” indicator for HIV infection [36], the social disposition that contextualizes or propagates syndemic among YMSM living with HIV in urban Bangkok may experience a broader scale of impact, resulting in an adverse health trajectory predating their HIV infections and prior to the social patterning of risky sexual behaviors [37].

We hypothesized that psychosocial adversities, including internalized HIV stigma, low social support, sex work, and low-income status, cluster with SAVA syndemic indicators (amphetamine use, binge drinking, intimate partner violence, homophobic bullying, and general bullying), thus increasing the risk of suicide in YMSM living with HIV in Bangkok. We also propose that these clusters might have a combined effect on suicidality through their interactions with depression, highlighting a potential syndemic effect. Moreover, we would expect a dose-response effect, by which the interacting effect of clustering adversities and depression increases as the grouping of clustering adversities increases in the following order: (1) SAVA only, (2) SAVA, low social support, HIV stigma; and (3) SAVA, low social support, HIV stigma, history of sex work, and low-income status. We used latent class analysis (LCA), a mixture modelling approach, to model the clustering of psychosocial adversities as latent classes. LCA identifies subgroups exhibiting specific combinations of co-occurring adversities, revealing diverging syndemic forms without assuming linear or additive effects, as exemplified by Lett et al. in modeling clustering substance use and depressive symptoms on suicidality [38]. Using causal inference methods, including directed acyclic graphs (DAGs) and propensity score weighting with more explicitly defined causal assumptions and robust inferences, we aimed to estimate the joint average exposure [treatment] effect (ATE) between clustering psychosocial adversities and depression on suicidality [39, 40]. Furthermore, we hypothesized that LCA models with different compositions of clustering psychosocial adversities might moderate the relationship between depression and suicidality differently, providing insight into whether potential depression treatment could be effective among subgroups with distinct patterns of clustering psychosocial adversities.

## Materials and Methods

### Participant Recruitment

A total of 214 participants were recruited into a 12-month prospective cohort study of young MSM (aged 15–29 years old) living with HIV in Bangkok, Thailand, with the assistance of community-based organization (CBO) partners, including the Poz Home Center Foundation and Rainbow Sky Association of Thailand (RSAT). The eligibility criteria included (1) male sex at birth, (2) ability to speak Thai, (3) Thai nationality, (4) having had anal intercourse with a man in the past 6 months, (5) living in Bangkok for at least 6 months, (6) age between 15 and 29 years, (7) consent to be followed up online every four months for a total period of 12 months (T1-T4), and (8) self-reported HIV status as living with HIV. Recruitment and baseline measurements (T1) began between January and February 2018, and the project staff recruited eligible participants by sending a URL that led to a web-based survey using the Qualtrics software. Upon completion of the survey, participants received 500 baht (15 USD) to compensate for their time. Participants were referred to mental health professionals based on their willingness and consent to discuss personal concerns, including substance use and violence. Informed consent was provided by all participants prior to enrollment, and the Mahidol University Institutional Review Board reviewed and approved all the study procedures (COA 2017/078.2803).

### Measures

#### Sociodemographics

Participants self-reported their age (in years), employment status, educational level, guardian’s or parents’ highest education, monthly income, sexual orientation, whether they had ever received money, goods, or other considerations (e.g., drugs and alcohol, mobile phones, mobile phone credits, clothes, bags, grades, or educational opportunities) in exchange for sex and time since HIV diagnosis.

#### Suicidality

Suicidality in our study was measured at baseline and repeated at T2 (4 months), T3 (8 months), and T4 (12 months) using the 4-item Suicidal Behaviors Questionnaire-Revised (SBQ-R) [41] adapted from a back-translated version [41, 42]. This tool evaluates a spectrum of suicidal behaviors through its items, which cover the following domains: lifetime suicidal ideation and attempts, frequency of suicidal ideation in the past year, threats to committing suicide, and perceived likelihood of future suicidal ideation. In our sample, the SBQ-R demonstrated satisfactory internal consistency, with a Cronbach’s alpha of 0.81, closely matching the scale’s application in the Thai population [41, 43]. Scores on the SBQ-R are cumulative, ranging from 3 to 18, and a predictive cutoff of 7 or higher has been established among university students in the Midwestern United States. Given that this cutoff has not been validated in the Thai population, we treated the SBQ-R score as a continuous variable. The purpose of the SBQ-R in this study was to gauge the degree of suicidal tendency as reported by the participants, rather than to determine a fixed predictive probability.

#### Psychosocial Adversity Indicators

*Depression* was assessed by participants’ self-reported past 7 day depression symptomology using the Center for Epidemiologic Studies Short Depression Scale (CESD-R) [44]. The scale consisted of 10 items. The total depression score was calculated by summing up all 10 items, with possible scores ranging from 0 to 30. A summed score of ≥ 10 indicates elevated depressive symptoms [45]. We found the internal consistency of this scale within the sample to be within the acceptable range (α = 0.686). The CESD-R, including its cut-off point, has been widely utilized in Southeast Asia and in research on Thai people [29, 46, 47]. *Internalized HIV stigma* was assessed using the 12-item short version of the HIV Stigma Scale [48]. Items were summed up to calculate the total HIV stigma score, with a possible range of 12–48. Among the participants, those who scored above the sample mean were categorized as having a high level of internalized HIV stigma and vice versa. The overall measure demonstrated adequate internal consistency (α = 0.88) and has been validated and utilized among young Thai people living with HIV [22, 49]. *Social support* was measured using the 12-item modified version of the Social Provision Scale (SPS) [50], which includes the domains of tangible support, attachment, nurturance, social integration, reassurance of worth, and guidance. The scale has a total score range of 12–48 with excellent internal consistency in our sample (α = 0.91), similar to its application in previous literature among Thai youth living with HIV [22]. *Intimate partner violence* was assessed by asking participants whether they had experienced the following behaviors in the past six months: hurting, hitting, slapping the body by a regular partner, casual sex partner, or male sex work partner; being forced to have sex by a regular partner or casual sex partner; being forced to have sex during sex work; and being fondled or having unwanted sexual contact against their will. Participants reporting any of the above experiences were categorized as having experienced intimate partner violence in the past six months. *Homophobic bullying* was assessed by asking the participants whether they had been bullied because of their sexual orientation or because they did not act like boys or men. *Amphetamine use* was assessed by asking how often participants had used the following drugs in the past six months: crystal meth/ice (inhaling/smoking), crystal meth/ice (injecting), and amphetamine (yaba/yama). Those reporting at least “once or twice“ in the past six months were categorized as amphetamine users. *Binge drinking* was assessed by asking the participants how often they consumed six or more drinks on one occasion. Those who reported weekly or more frequent alcohol use were categorized as binge drinkers. Only baseline measurements were used for these clustering adversity indicators, because the clustering of psychosocial adversities likely began during early adolescence and propagated beyond HIV serostatus conversion.

### Data Analysis

Participants were characterized by covariate proportions and their categorical means and standard deviations of suicidality scores at baseline and 12-month follow-up (T4), and proportional mean differences by Wilcoxon rank-sum tests. A bias-adjusted, 3-step latent class analysis (LCA) approach was used to classify participants into distinct clusters based on psychosocial adversity indicators using the poLCA package in R, which estimates LCA model parameters through maximization-like Newton-Raphson algorithms [51–53]. Three LCA models were tested: Model 1 included SAVA indicators only: homophobic bullying, general bullying, intimate partner violence (IPV), amphetamine use, and binge drinking; Model 2 included SAVA indicators from Model 1 and internalized HIV stigma and low social support; and Model 3 included all variables from Model 2 and a history of sex work and low-income status. LCA class enumeration was guided by fit indices with 10,000 iterations and 100 starting values while including covariates as distal outcomes to address classification uncertainty and differential item functioning [54].

To estimate the joint average treatment [exposure] effect (ATE) of clustering psychosocial adversities and depression, we used a counterfactual causal-inference framework. For each of the three latent class models, directed acyclic graphs (DAGs) were constructed and statistically validated using the daggity package in R to inform the construction of propensity score weights for marginal structural modeling using weighted generalized estimating equations (GEEs), accounting for confounding by latent class assignment, depression status, and loss-to-follow-up [55–57].

We fit propensity score models with the corresponding minimal sufficient adjustment set informed by DAGs to derive two sets of overlap weights (OW) (latent class and depression) and inverse probability censoring weights (IPCW) for loss-to-follow-up [40, 55, 58]. Overlap weights are more consistent with health disparity research among minority populations, as they upweight participants with propensity scores around 0.5, thus inferring a counterfactual pseudo-population (the weighted sample) with a similar probability of being exposed to multiple psychosocial adversities rather than those most or least likely to be exposed by inverse probability weighting (IPW) [40, 58, 59]. IPW is more appropriate for censoring patients by upweighting those most likely to be lost-to-follow-up [58]. Weights were additionally adjusted for latent class posterior probabilities to account for classification uncertainty [53, 60], assessed by adjusted covariates’ standardized mean differences (SMD), visually diagnosed using box and density plots, and trimmed to remove outliers at the extreme percentiles. Weighted GEEs with robust variance estimators were utilized to estimate ATEs, while accounting for clustered variance by latent class if the intraclass correlation coefficients (ICCs) between the latent class variable and suicidality were above a moderate level (> 0.20). Models with main effects and interaction terms were compared using the QIC statistics and a z-distribution-based score test. To assess effect modification, latent classes were stratified to estimate the stratum-specific ATEs of depression on suicidality, and the estimated marginal means (package emmeans in R) were calculated to estimate the simple effects of quantifying counterfactual treatment on depression among participants with different pre-existing clustering psychosocial adversities. Uncertainty parameters were adjusted for multiple testing using Tukey’s method [61]. All analyses were performed in R using R Studio (version 2024.4.2.764) [62].

### Sensitivity Analysis

Untrimmed weighted GEE with overlap weights, untrimmed weighted GEE with inverse probability weights, unweighted GEE adjusted for confounding only, unweighted GEE adjusted for confounding, and baseline suicidality were conducted for the final LCA model (Model 3) to evaluate its robustness against modeling misspecifications.

## Results

### Participant Characteristics

A total of 214 Thai YMSM living with HIV were enrolled in the cohort study and had an attrition rate of 11.2% (*N* = 190) at T4 (12-month). Sociodemographic characteristics of the participants are presented in Table 1. One-fifth (20.6%) of the participants were aged 15–25 years; a quarter (24.8%) had secondary education or below; the majority of the participants’ guardians had only completed secondary education (76%); a quarter (25.7%) were currently unemployed; and one-fifth (20.6%) had an income of 5000 Baht (∼ 150 USD) or less, while most participants (95%) were identified as gay. Less than half (46%) had a regular male partner in the past 12 months, almost two-thirds (59%) had been diagnosed with HIV for more than a year, and a quarter had ever provided sex work (27%). About one-third of the participants (31% and 34%, respectively) had experienced homophobic and general bullying during their lifetime, while over half (55%) were categorized as having a high level of internalized HIV stigma (above sample median). About a quarter (25%) were categorized as having a low level of social support (cut-off at the 1st quantile), 14% reported intimate partner violence, 45% reported a clinically significant level of depressive symptoms, 12% reported amphetamine use in the past six months, and 13% reported binge drinking in the past six months. There were significant mean differences (Table 2) in baseline suicidality scores within the levels of employment, income, homophobic bullying, general bullying, intimate partner violence, internalized HIV stigma, amphetamine use, and depression.

**Table 1:** Sociodemographic and psychosocial factors and their associations with suicidality among young HIV + Thai MSM (*N* = 214)

			Suicidality at baseline (T1)			Suicidality at 12 months (T4)		
Characteristics	N	(%)	Mean	(SD)	P	Mean	(SD)	P
Age								
15–25	44	(20.56)	5.80	(3.59)	0.173	4.98	(2.92)	0.005
26–29	170	(79.44)	5.08	(3.14)		3.89	(2.07)	
Education								
Secondary or below	53	(24.77)	5.17	(3.04)	0.557	4.09	(2.39)	0.665
Tertiary or above	161	(75.23)	5.24	(3.31)		4.12	(2.29)	
Guardian’s education								
Secondary or below	163	(76.17)	4.93	(2.97)	0.017	3.97	(2.18)	0.055
Tertiary or above	51	(23.83)	6.18	(3.87)		4.53	(2.65)	
Employment								
Full-time	116	(54.21)	5.03	(3.01)	0.049	4.05	(2.31)	0.090
Part-time	43	(20.09)	4.63	(2.71)		3.60	(1.39)	0.090
Unemployed	55	(25.70)	6.11	(3.90)		4.65	(2.76)	
Income								
5000 Baht or below	44	(20.56)	6.27	(3.85)	0.041	3.98	(2.01)	0.243
5001-15,000 Baht	100	(46.73)	5.11	(3.10)		4.64	(2.62)	0.243
15,001 Baht or above	70	(32.71)	4.73	(2.90)		3.96	(2.46)	
Sexual Orientation								
Gay	203	(94.86)	5.26	(3.28)	0.647	4.09	(2.32)	0.460
Bisexual	11	(5.14)	4.64	(2.50)		4.45	(2.21)	
Regular male partner in past 12 months								
No	115	(53.74)	5.46	(3.19)	0.048	4.39	(2.51)	0.020
Yes	99	(46.26)	4.95	(3.29)		3.78	(2.02)	
Recent HIV diagnosis (less than a year)								
No	87	(40.65)	5.44	(3.49)	0.574	4.00	(2.08)	0.588
Yes	127	(59.35)	5.08	(3.06)		4.26	(2.59)	
Ever provided sex work								
No	157	(73.36)	5.10	(3.04)	0.486	4.06	(2.28)	0.400
Yes	57	(26.64)	5.56	(3.73)		4.27	(2.40)	
Homophobic bullying, ever								
No	148	(69.16)	4.74	(2.99)	< 0.001	3.96	(2.29)	0.022
Yes	66	(30.84)	6.30	(3.52)		4.44	(2.33)	
General bullying, ever								
No	142	(66.36)	4.58	(2.63)	< 0.001	3.97	(2.14)	0.172
Yes	72	(33.64)	6.50	(3.90)		4.38	(2.58)	
Intimate partner violence, past 6 months								
No	185	(86.45)	5.09	(3.26)	0.004	4.10	(2.34)	0.594
Yes	29	(13.55)	6.10	(3.00)		4.18	(2.13)	
Amphetamine use								
No	188	(87.85)	4.91	(2.97)	< 0.001	4.08	(2.24)	0.955
Yes	26	(12.15)	7.46	(4.18)		4.32	(2.83)	
Binge drinking								
No	186	(86.92)	5.24	(3.30)	0.693	4.18	(2.35)	0.369
Yes	28	(13.08)	5.14	(2.88)		3.67	(2)	
Depression (CESD-R)								
Mean (SD)	12.6	(5.38)						
No (CESD-*R* < 16)	117	(54.67)	4.00	(2.08)	< 0.001	3.37	(1.23)	< 0.001
Yes (CESD-*R* ≥ 16)	97	(45.33)	6.70	(3.75)		5.01	(2.93)	
Internalized HIV stigma								
Mean (SD)	33.5	-6.98						
Above the sample mean	155	(72.43)	5.07	3.08	0.502	3.97	(2.18)	0.121
Below the sample mean	59	(27.57)	5.63	3.63		4.48	(2.60)	
Low social support								
Mean (SD)	38.0	(5.56)						
Above the sample first quantile	160	(74.77)	5.08	(3.17)	0.266	3.94	(2.17)	0.044
Below the sample first quantile	54	(25.23)	5.67	(3.42)		4.62	(2.63)	
Loss to follow up								
No	190	(88.79)	4.83	3.07	0.441	3.70	(1.89)	0.547
Yes	24	(11.21)	5.27	3.26		4.13	(2.33)	

*Wilcoxon rank-sum test

**Table 2:** Item response probabilities of latent class models quantifying the clustering of psychosocial adversities among Thai MSM living with HIV (*N* = 214)

	Clustering of psychosocial adversities		
	Class 1 - low	Class 2 - moderate	Class 3 - high
Model 1: SAVA syndemics			
n, %	*n* = 87, 40.7%	*n* = 41, 19.2%	*n* = 86, 40.2%
Sum score, mean	1.33	1.83	4.00
Suicidality at baseline, mean	3.07	7.51	6.31
Suicidality at T4, mean	3.38	5.35	4.27
Homophobic bullying	0.016	0.023	0.745
General bullying	0.009	0.119	0.765
Intimate partner violence	0	0.026	0.324
Amphetamine use	0.021	0.198	0.168
Binge drinking	0.117	0.050	0.192
Model 2: SAVA + internalized HIV stigma + low social support			
n, %	*n* = 83, 34.4%	*n* = 64, 30.0%	*n* = 67, 31.3%
Sum score, mean	1.31	2.03	4.42
Suicidality at baseline, mean	3.06	6.73	6.46
Suicidality at T4, mean	3.39	4.83	4.30
Homophobic bullying	0.398	0.512	0.719
General bullying	0	0.121	0.772
Intimate partner violence	0	0.167	0.811
Amphetamine use	0	0.027	0.363
Binge drinking	0.002	0.207	0.156
Internalized HIV stigma	0.127	0.052	0.207
Low social support	0.242	0.160	0.346
Model 3: SAVA + internalized HIV stigma + low social support + sex work + low income (social class)			
n, %	*n* = 76, 35.5%	*n* = 100, 46.7%	*n* = 38, 17.8%
Sum score, mean	1.25	1.74	3.74
Suicidality at baseline, mean	3.08	6.42	6.40
Suicidality at T4, mean	3.41	4.64	4.44
Homophobic bullying	0	0	0.637
General bullying	0	0.051	0.675
Intimate partner violence	0	0	0.280
Amphetamine use	0.026	0.099	0.195
Binge drinking	0.105	0.093	0.163
Internalized HIV stigma	0.160	0.243	0.367
Low social support	0.253	0.123	0.302
Sex work	0.170	0.120	0.388
Low income	0.482	1.000	0.674

### Latent Class Analysis

Fit indices from LCA indicate that a 3-class model provides the optimal fits for LCA models 1, 2, and 3, finalized by the Lo-Mendell-Rubin Likelihood Ratio Test (LMR-LRT) (Supplementary Table 1), showing that a 4-class model does not provide more information than a 3-class model (*p* > 0.05) but is significantly more informative than a 2-class model (*p* < 0.05). The relative entropy (RE) values indicated excellent (0.94), borderline acceptable (0.68), and good (0.82) discrimination in classifying participants in LCA models 1, 2, and 3, respectively.

The three LCA models identified three latent classes characterized by varying item response probabilities of adversity indicator endorsements, interpretable as the prevalence among participants assigned to each latent class. Converging themes across all models showed that latent class 3 consistently captured participants with the highest endorsements in adversity indicators, and latent class 2 captured those with moderate endorsements, with class 1 having the least (Table 2). However, across all LCA models, the mean suicidality scores at baseline and T4 were highest among participants assigned to Class 2, except for baseline suicidality in LCA Model 3 in Class 2. All participants in Class 2 of LCA Model 3 endorsed a low-income status with moderate internalized HIV stigma, but the least endorsement was for low social support and a history of sex work. No crude linear relationship was observed between summed scores (for all adversity indicators) and suicidality.

### Marginal Structural Models

All marginal structural models estimated by the weighted GEE accounted for clustering variance by the latent class variable due to the ICC value between the latent class variable and suicidality at baseline, computed as 0.41, 0.36, and 0.32, respectively, for LCA Model 1,2 and 3. Supplementary Figs. 1-3a, b, and c show the DAG, boxplot, and density diagnostics, respectively, demonstrating that the propensity score weights had adequate overlap, upholding the positivity assumption of no zero probability of being assigned to either treatment group (by latent class and depression status) across LCA models 1, 2, and 3. Weights were subsequently trimmed by extreme percentiles between 0.01 and 0.05 and 0.99 − 0.95 to reduce outlier bias. Supplementary Table 2 shows that the standard mean differences (SMD) of covariates were reduced after combined weighting (OWs and IPW) in LCA model 3, supporting a tendency towards exchangeability upheld by weighted covariate balance. Supplementary Table 3 shows the summary statistics comparing model fits between the main effects only and interaction-weighted GEE models. All interaction models had a better model fit than the main effect-only models. Supplementary Table 4 presents the main effects of the models alone.

### Estimations of the Average Exposure Effects for Suicidality at Baseline and One Year after

Table 3 shows the marginal structural models for estimating the synergistic or joint average treatment effect (ATE) of exposure to a given adversity cluster (latent class) versus the reference group (class 1, low adversity cluster) and depression on suicidality (departure from additivity), both cross-sectionally at baseline and longitudinally over a one-year period. In LCA Model 1, when only SAVA syndemic indicators were included, participants in Class 3 (highest endorsement of adversities) had significantly higher suicidality than did those in Class 1 (lowest endorsement). While there were significant synergistic effects between Class 3 and depression on suicidality at baseline, it did not significantly affect suicidality at 12-month; however, the interaction between Class 2 and depression became significant at T4, while it was not significant at baseline. In LCA Model 2, after including internalized HIV stigma and low social support, the interaction between Class 3 and depression was not significant at baseline, but both classes 2 and 3 interacted significantly with depression in suicidality at T4. For LCA Model 3, which further included low-income status and history of sex work, both Class 2 and Class 3 interactions with depression at baseline and suicidality at T4 were significant. The effect sizes of the interactions corresponded to the extent of clustering adversity (class 3 > class 2). Table 4 shows the stratum specific ATE of depression on suicidality at baseline and T4 to be interpreted as – had everyone with the given pattern of clustering adversities in class 3 of LCA Model 3 been treated for depression at baseline, their reported suicidality score would have been reduced on average by -3.69 (95% CI: -6.12, -1.27) units after a one-year period; and similarly, by -1.32 (95% CI: -2.31, − 0.32) units after a one-year period, had everyone with the given pattern of clustering adversities in class 2 of LCA Model 3 been treated for depression at baseline.

**Table 3:** Synergistic effects of clustering psychosocial adversities and depression on suicidality

	Lifetime suicidality at baseline			Past 4 month suicidality at T4		
	*β*_*ow*_	95% CI_*ow*_	*P*_*ow*_	*β*_*ow*_	95% CI_*ow*_	*P*_*ow*_
LCA Model 1: SAVA syndemic (homophobic bullying, general bullying, intimate partner violence, amphetamine use, binge drinking)						
Class 1 (intercept)	3.03	3.00–3.07	**< 0.001**	3.26	3.00–3.51	**< 0.001**
Class 2	3.48	2.29–4.66	**< 0.001**	-0.04	-0.43–0.36	0.852
Class 3	1.63	1.01–2.24	**< 0.001**	0.06	-0.33–0.44	0.777
Depression	0.07	-0.06–0.21	0.293	0.06	-0.35–0.48	0.766
Class 2 × depression	1.42	-0.29–3.12	0.105	3.19	1.42–4.96	**< 0.001**
Class 3 × depression	2.31	1.01–3.61	**0.001**	1.02	-0.31–2.34	0.132
LCA Model 2: SAVA syndemic + internalized HIV stigma + low social support						
Class 1 (intercept)	3.04	3.00–3.07	**< 0.001**	3.30	3.04–3.56	**< 0.001**
Class 2	1.32	0.64–2.00	**< 0.001**	-0.17	-0.47–0.14	0.293
Class 3	3.06	2.12–4.00	**< 0.001**	0.11	-0.34–0.56	0.641
Depression	0.03	-0.10–0.16	0.656	0.08	-0.41–0.56	0.753
Class 2 × depression	3.14	1.66–4.62	**< 0.001**	1.38	0.28–2.47	**0.013**
Class 3 × depression	1.32	-0.07–2.71	0.062	1.68	0.04–3.32	**0.044**
LCA Model 3: SAVA syndemic + internalized HIV stigma + low social support + sex work + low income						
Class 1 (intercept)	3.03	3.00–3.06	**< 0.001**	3.65	3.07–4.24	**< 0.001**
Class 2	1.53	0.59–2.47	**0.001**	-0.48	-1.09–0.13	0.126
Class 3	3.08	1.85–4.31	**< 0.001**	-0.63	-1.22 – -0.04	**0.036**
Depression	0.01	-0.04–0.06	0.666	-0.16	-1.14–0.81	0.745
Class 2 × depression	2.50	1.12–3.88	**< 0.001**	1.48	0.09–2.87	**0.037**
Class 3 × depression	3.61	1.12–6.09	**0.005**	3.86	1.24–6.47	**0.004**

*β*_*ow*_, CI_*ow*_, *P*_*ow*_ : coefficients, confidence interval and *p*-value from generalized estimating equation estimating the average exposure [treatment] effects (ATE) from marginalized structural model by overlap weights (OW) with robust standard errors (sandwich) and clustering by latent class variable

**Table 4:** Stratum specific average exposure effect and effect modification of depression by latent class on suicidality

	Lifetime suicidality at baseline			Past 4 month suicidality at T4		
	*β*_*ow*_	95% CI_*ow*_	*P*_*ow*_	*β*_*ow*_	95% CI_*ow*_	*P*_*ow*_
LCA Model 1: SAVA syndemic (homophobic bullying, general bullying, IPV, amphetamine use, binge drinking)						
Class 1: n1 = 87 / n2 = 81						
(Intercept)	3.04	3.00–3.07	**< 0.001**	3.25	3.01–3.50	**< 0.001**
Depression	0.07	-0.08–0.22	0.338	0.06	-0.36–0.47	0.796
δ-depression	-0.07	-0.21–0.06	0.293 ^a^	-0.06	-0.48–0.35	0.766 ^a^
Class 2: n1 = 41 / n2 = 37						
(Intercept)	6.53	5.35–7.72	**< 0.001**	3.18	2.89–3.47	**< 0.001**
Depression	1.43	-0.19–3.06	0.084	3.24	1.62–4.86	**< 0.001**
δ-depression	-1.49	-3.19–0.21	0.086^a^	-3.26	-4.98 – -1.53	**< 0.001**
Class 3: n1 = 86 / n2 = 82						
(Intercept)	4.56	3.91–5.21	**< 0.001**	3.29	3.02–3.57	**< 0.001**
Depression	2.47	1.14–3.80	**< 0.001**	1.09	-0.23–2.41	0.106
δ-depression	-2.38	-3.68 – -1.09	**< 0.001** ^**a**^	-1.08	-2.34–0.18	0.092 ^a^
LCA Model 2: SAVA syndemic + internalized HIV stigma + low social support						
Class 1: n1 = 83 / n2 = 77						
(Intercept)	3.06	3.00–3.12	**< 0.001**	3.38	3.04–3.73	**< 0.001**
Depression	0.01	-0.13–0.14	0.973	0.02	-0.59–0.64	0.940
δ-depression	-0.03	-0.16–0.1	0.656 ^a^	-0.08	-0.56–0.41	0.753 ^a^
Class 2: n1 = 64 / n2 = 60						
(Intercept)	5.95	4.65–7.26	**< 0.001**	3.53	3.06–4.01	**< 0.001**
Depression	1.18	-0.51–2.88	0.172	2.08	0.84–3.32	**0.001**
δ-depression	-1.35	-2.73–0.03	0.055 ^a^	-1.76	-3.32 – -0.19	**0.028** ^**a**^
Class 3: n1 = 67 / n2 = 63						
(Intercept)	4.25	3.62–4.88	**< 0.001**	3.14	2.95–3.34	**< 0.001**
Depression	3.16	1.92–4.39	**< 0.001**	1.65	0.88–2.42	**< 0.001**
δ-depression	-3.17	-4.65 – -1.69	**< 0.001** ^a^	-1.45	-2.43 – -0.48	**0.004** ^**a**^
LCA Model 3: SAVA syndemic + internalized HIV stigma + low social support + sex work + low income						
Class 1: n1 = 76 / n2 = 71						
(Intercept)	3.07	3.00–3.13	**< 0.001**	3.43	3.06–3.79	**< 0.001**
Depression	0.04	-0.12–0.20	0.602	-0.10	-0.72–0.51	0.739
δ-depression	-0.01	-0.06–0.04	0.666 ^a^	0.16	-0.81–1.14	0.745 ^a^
Class 2: n1 = 100 / n2 = 93						
(Intercept)	4.55	3.67–5.42	**< 0.001**	3.28	3.01–3.56	**< 0.001**
Depression	2.51	1.31–3.71	**< 0.001**	1.58	0.84–2.33	**< 0.001**
δ-depression	-2.52	-3.9– -1.14	**< 0.001** ^**a**^	-1.32	-2.31 – -0.32	**0.009** ^**a**^
Class 3: n1 = 38 / n2 = 36						
(Intercept)	5.39	4.42–6.36	**< 0.001**	3.37	2.83–3.91	**< 0.001**
Depression	2.76	0.50–5.02	**0.017**	3.28	1.32–5.24	**< 0.001**
δ-depression	-2.62	-6.10 – -1.13	**0.004** ^**a**^	-3.69	-6.12 – -1.27	**0.003** ^**a**^

*β*_*ow*_, CI_*ow*_, *P*_*ow*_ : coefficients, confidence interval and *p*-value from generalized estimating equation (GEE) estimating the average exposure [treatment] effects (ATE) from marginalized structural model by overlap weights (OW) with robust standard errors (sandwich).

n1 = number of observations by strata of latent class in baseline suicidality outcome model

n2 = number of observations by strata of latent class in suicidality at T4 outcome model

^**a**^*P*-value from Z ratio distribution against the null hypothesis that the difference between *β*_*ow*_ (depression = 0) - *β*_*ow*_ (depression = 1) equals 0 after adjusted for multiple comparison by the Tukey method

### Sensitivity Analysis

Consistent results were obtained for the untrimmed weighted GEE with overlap weights, untrimmed weighted GEE with inverse probability weights, unweighted GEE adjusted for confounding only, unweighted GEE adjusted for confounding, and baseline suicidality in the estimation of ATE in LCA Model 3 (Supplementary Table 4). However, the synergistic effect between class 2 (moderate adversity clustering) and depression was only borderline significant for the weighted GEE with untrimmed overlap weights and unweighted GEE models^1^.

## Discussion

This study is the first to investigate the syndemic effect of clustering psychosocial adversities and depression on suicidality among HIV-positive young men who have sex with men (YMSM) using a combination of latent class analysis and causal inference methods. Our findings provide evidence supporting a synergistic effect (departure from additivity) between clustering psychosocial adversities and depression on suicidality among YMSM living with HIV in Bangkok. This joint contribution to suicidality offers empirical evidence suggesting that effective treatment for depression would be beneficial in reducing suicidal tendencies in this population, regardless of patterns of clustering psychosocial adversities as indicated by latent class memberships. While a multicomponent intervention addressing both clustering psychosocial adversities and depression would be ideal and would yield the greatest reduction in suicidality, our results indicate that currently available depression treatments alone could provide substantial benefits in reducing the projected suicidality among YMSM living with HIV in Bangkok [16, 19]. These findings highlight the importance of prioritizing accessible and effective depression interventions for this vulnerable population, even in the absence of effective multicomponent programs targeting the full range of psychosocial adversities they might face [15, 17, 18]. An integrated model for mental health and HIV health services can significantly reduce suicidality among YMSM living with HIV in Bangkok [19].

More specifically, we demonstrated that groups of clustering adversities interacted differently with depression to jointly contribute to suicidality at baseline and one-year preceding baseline. When internalized HIV stigma was conceptualized as a confounder between clustering adversity (LCA model 1) and suicidality reported one year after baseline, and low social support as a downstream mediator between internalized HIV stigma and baseline depression; the total effect of the hypothesized SAVA latent class included the following downstream mediators: homophobic and general bullying, IPV, binge drinking, and amphetamine use, and the direct effect of SAVA latent class on suicidality. In this conceptualization, participants belonging to the latent class with the highest prevalence of clustering adversities (latent class 3 of Model 1) did not interact with depression to predict suicidality one year later. This is most likely due to the theoretical linkage that internalized HIV stigma (and low social support to a lesser extent) should have been included as a downstream or total effect of the hypothesized syndemic latent class together with SAVA indicators rather than adjusted as a confounder. This hypothesized causal structure (DAG from Supplementary Fig. 1a) attenuated the total effect between SAVA and suicidality, and internalized HIV stigma (and low social support) as downstream causal mediators of SAVA. The lack of a synergistic effect with depression to predict suicidality longitudinally indicates that SAVA-only adversity indicators, despite having clustering relationships, do not constitute a syndemic effect according to the most recent clarification of syndemic theory [8, 18]. Additionally, none of the adversity indicators interacted singly with depression to increase suicidality despite being bivariately associated with suicidality. This is consistent with Brown et al. ’s finding that SAVA indicators were independently or additively associated with suicide-related outcomes but did not interact to further elevate suicide-related outcomes [63].

In the second LCA model, internalized HIV stigma and low social support were included along with other SAVA indicators as downstream mediators of the latent class variable. In this conceptualization, there is evidence of a synergistic effect between a high level of clustering adversities (class 3) and depression in predicting suicidality one year after the baseline. However, there is no evidence of a synergistic effect in the cross-sectional model. One interpretation drawn from the minority stress theory is that internalized HIV stigma and a low level of social support could be considered proximal stressors that interact with other proximal stress processes, such as depression, which were captured at the moment of measurement at baseline (neither scale has a recall period) and may not correspond to their prior values in predicting suicidality at baseline [20, 21, 64]. In other words, it takes time for the levels of internalized HIV stigma and social support to interact with depression in affecting suicidality; however, their prior values that predict suicidality one year later were captured at baseline. This interpretation is consistent with a longitudinal study among Canadian young adults aged 19–20, in which a high level of social support was postulated to buffer elevated proximal stressors, such as depression, and reduce the risk for suicide-related outcomes during the one-year study period [65].

In the third LCA model, we assumed a wider range of total exposure effects (all the green arrows in Supplementary Fig. 3a) with a hypothesized syndemic process that includes social categories such as low income and history of sex work, assuming an economically reinforced social hierarchy to reflect social reality, despite having equal educational opportunities through national policies and birth (parent/guardian’s education), and subsequent employment status. Sexual minorities in Thailand face unequal levels of social treatment in all spheres of their daily lives (e.g., school, work, home, and other public spaces) [31, 33]. After including these social categories, depression interacted with clustering adversities to further increase suicidality at both moderate and high clustering levels (Classes 2 and 3), cross-sectionally, and following the one-year study period. The effect sizes of the synergistic effects were more pronounced than those of prior adversity clusters (LCA models 1 and 2), and were consistent with the dose-response pattern of previously reported clustering adversities [9, 13, 66]. This result is robust against weighting methods (IPW), unweighted GEE adjusted by DAG-informed confounders, and confounding factors including baseline suicidality (Supplementary Table 5).

The findings of our final model align with the recent discourse on syndemic theory, which posits that contextual factors such as sex work engagement and inequitable income distribution are clustered with psychosocial adversities, including SAVA indicators, internalized HIV stigma, and low social support levels [8, 30, 67]. A syndemic pattern has also been observed among African-American MSM living with HIV in the US [36]. These clustering adversity patterns produced magnified synergistic effects with depression, predicting increased suicidality levels more than the summed (or additive) average total exposure effects. This highlights a possible indication that multiple social categories, such as low income and sex work, might have promoted such clustering; for example, using crystal meth with sex work clients [68] and/or the potential for adverse interaction with depression, such as IPV by crystal meth using sex work clients [69], widening the disparity in suicidality among YMSM living with HIV in Bangkok [16, 30, 70]. Our observations are consistent with cross-sectional data showing that Thai LGBT adults residing in high-poverty regions with co-occurring experiences of victimization, loneliness, and stress are independently associated with suicide ideation [26]. Despite the conceptual relevance of contextual processes, the precise adversity that promotes initial clustering (e.g., bullying vs. sex work) remains uncertain. A more nuanced understanding of the social marginalization process on how aggregated or cumulative distal stressors, such as being consistently bullied at school, workplace, or homes, and the chronological order of these clustering adversities underlined by sex work engagement and inequitable earned income levels, and how they either increase the probability of adversity clustering or interact with adverse mental health states, worsens well-being [71]. This information can inform more precisely designed interventions or public policies to intersect the disparity-generating syndemic processes.

### Limitations

The interpretation of the results of this study had some limitations. The HIV status was self-reported without further ascertainment; therefore, it may not have accurately reflected the actual HIV serostatus of the included participants. Our sample of HIV-positive YMSM recruited via the CBO may not be representative of the underlying population. CBO-engaged HIV-positive YMSM may have a lower prevalence of clustering adversities and suicidality than those who are not engaged, potentially underestimating the average exposure effects (ATE) between the latent class and suicidality. The use of retrospective self-reports is subject to recall bias, leading to the misclassification of psychosocial variables, especially those with longer recall periods such as ever experienced homophobic bullying, which may underestimate the relationship between psychosocial variables and suicidality. In addition, binge drinking was measured as having six or more drinks in one setting, which is inconsistent with the dominant literature on classifying binge drinking (five or more), leading to a possible misclassified underestimation, favoring the null hypothesis. The accuracy of our estimated average exposure effect depends on the correctly specified LCA and propensity score models; misspecification of either model can lead to biased results. However, we used bias adjustment in LCA modelling, including covariates as distal outcomes and accounting for LCA model uncertainty, to incorporate posterior probability in propensity score weighting, which may attenuate bias due to differential item functioning and latent class misclassification [52–54]. The use of additional weighting methods (OW and IPW) and unweighted regression provided robustness against propensity score-weighting misspecifications. Although our sample size lies at the far end of the optimal range for adequate class separation in LCA, the relative entropy value of 0.82 in model 3 demonstrates adequate class separation [72]. The consistency of the stable unit treatment assumption is least likely to uphold because the latent class assignment may change depending on the number of parameters and parameter values of the classification model. The potential treatment for clustering adversities defined by the latent class is not well-defined [53]. However, our discussion focused mainly on the potential treatment of depression, as demonstrated by the effect of modification of depression on suicidality by the latent class. Visualization of our modeled weights demonstrates sufficient support for the positivity assumption; however, we cannot ascertain exchangeability (no unmeasured confounding) despite the weighting method reducing covariate imbalances between latent class variables. Finally, our analysis captured individuals who had attempted suicide and survived, which may have different characteristics from those who did not survive.

### Conclusion

Our study provides novel evidence of a synergistic relationship among clustering psychosocial adversities, depression, and suicidality among young men who have sex with men living with HIV in Bangkok, indicating a syndemic effect. The magnified joint effects on suicide risk highlight the urgent need to implement integrated mental health and HIV care services with a focus on accessible and effective depression treatment to reduce suicidality in this vulnerable population, even among subgroups with the highest clustering of psychosocial adversities, including those with a history of sex work and low-income status. Although multicomponent interventions targeting the full range of psychosocial challenges would be ideal, our findings indicate that even standalone depression treatment could yield substantial benefits in the absence of effective multicomponent interventions. Further research should explore the underlying social marginalization processes that contextualize or initiate and promote adversity clustering and elucidate the temporal sequencing of events to inform more precisely designed and targeted syndemic interventions. Overall, this study underscores the vital need to prioritize mental health within HIV care to mitigate the disproportionate burden of suicidality among young men who have sex with men living with HIV in Bangkok.

## Electronic supplementary material

Below is the link to the electronic supplementary material.

Supplementary material 1

## Data Availability

The datasets generated and analyzed during the current study are not publicly available but are available from the corresponding author on reasonable request.

## Footnotes

1 To encourage reproducibility and methodological exchange among health equity researchers, R codes for this manuscript’s analysis are available on GitHub repository (for enquiries, email: hoc307@gmail.com): https://github.com/hoc307/project_secondary.git.
