# Supplementary material for: A Syndemic Clustering of Adversities on Suicide Risk among YMSM Living with HIV in Bangkok: A Causal Latent Class Analysis
Source: AIDS Behav. 2025 Jan 9;29(2):420–34. doi: 10.1007/s10461-024-04516-7 (PMC11813946; doi:10.1007/s10461-024-04516-7)
Supplement: Supplementary file 1 — Supplementary material 1 [file 10461_2024_4516_MOESM1_ESM.docx]

| Supplementary Table 1. Fit and diagnostic indices for latent class enumeration | | | | | | | | | |
| --- | --- | --- | --- | --- | --- | --- | --- | --- | --- |
| LCA model 1: SAVA syndemics (homophobic bullying, general bullying, intimate partner violence, amphetamine use, binge drinking) | | | | | | | | | |
| Class | Npar | LL | BIC | aBIC | CAIC | AWE | RE | LMR-P | *SCP* |
| 2 | 14 | -445.25 | 965.63 | 921.27 | 979.63 | 1082.75 | 0.80 | <0.001 | 0.42 |
| 3 | 23 | -437.14 | 997.70 | 924.82 | 1020.70 | 1190.11 | 0.85 | <0.001 | 0.10 |
| 4 | 32 | -447.84 | 1067.40 | 966.00 | 1099.40 | 1335.11 | 0.94 | 1.00 | 0.00 |
| 5 | 41 | -473.31 | 1166.63 | 1036.71 | 1207.63 | 1509.63 | 0.98 | 1.00 | 0.00 |
| 6 | 50 | -445.25 | 1158.80 | 1000.37 | 1208.80 | 1577.10 | 0.92 | <0.001 | 0.00 |
|  | | | | | | | | |  |
| LCA model 2: SAVA syndemics + internalized HIV stigma + low social support | | | | | | | | | |
| Class | Npar | LL | BIC | aBIC | CAIC | AWE | RE | LMR-P | *SCP* |
| 2 | 18 | -685.94 | 1468.46 | 1411.43 | 1486.46 | 1619.05 | 0.80 | <0.001 | 0.37 |
| 3 | 29 | -674.37 | 1504.36 | 1412.46 | 1533.36 | 1746.97 | 0.68 | <0.001 | 0.32 |
| 4 | 40 | -681.95 | 1578.53 | 1451.78 | 1618.53 | 1913.17 | 0.90 | 1.00 | 0.00 |
| 5 | 51 | -710.80 | 1695.26 | 1533.66 | 1746.26 | 2121.93 | 0.95 | 1.00 | 0.00 |
| 6 | 62 | -685.94 | 1704.57 | 1508.10 | 1766.57 | 2223.26 | 0.92 | 0.00 | 0.00 |
|  | | | | | | | | | |
| LCA model 3: SAVA syndemics + HIV stigma + low social support + transactional sex + low income | | | | | | | | | |
| Class | Npar | LL | BIC | aBIC | CAIC | AWE | RE | LMR-P | *SCP* |
| 2 | 25 | -936.20 | 2006.54 | 1927.32 | 2031.54 | 2215.69 | 0.80 | <0.001 | 0.40 |
| 3 | 41 | -917.62 | 2055.25 | 1925.33 | 2096.25 | 2398.25 | 0.82 | <0.001 | 0.19 |
| 4 | 57 | -967.59 | 2241.04 | 2060.42 | 2298.04 | 2717.90 | 1.00 | 1.00 | 0.08 |
| 5 | 73 | -972.22 | 2336.15 | 2104.83 | 2409.15 | 2946.87 | 0.99 | 1.00 | 0.04 |
| 6 | 89 | -966.22 | 2410.02 | 2128.00 | 2499.02 | 3154.59 | 1.00 | <0.001 | 0.00 |
| Npar: number of parameters estimated.  LL: log likelihood ratio measures the model fit, a higher LL indicates a better fit.  BIC: Bayesian Information Criteria penalizes model complexity, a more parsimonious model has lower BIC.  aBIC: sample-size adjusted BIC with lower penalizing score for smaller sample sizes.  CAIC: Consistent Akaike Information Criteria penalizes complex models more severely than BIC.  AWE: Approximate Weight of Evidence Criteria provides joint indication of class separation and model fit  RE: Relative Entropy diagnoses how well the model assign participants to each latent class with a commonly used cut off at 0.70 indicating optimal class separation.  LMR-P: p-value from Lo-Mendell-Rubin-Likelihood Ratio Test comparing k vs k-1 number of classes.  SCP: smallest class proportion, the higher the better. | | | | | | | | | |

| Supplementary Table 2. Standard mean difference of covariates by LCA model 3 (SAVA + social factors) | | | |
| --- | --- | --- | --- |
|  | Standardized mean difference (SMD) | | |
|  | Unweighted | Combined weights– baseline | Combined weights – T4 |
| Age | 1.509 | 0.106 | 0.428 |
| Education | 0.294 | 0.052 | 0.133 |
| Employment | 0.654 | 0.252 | 0.617 |
| Regular partner | 0.396 | 0.152 | 0.195 |
| Time since HIV diagnosis | 0.170 | 0.129 | 0.105 |
| Depression | 1.076 | 0.188 | 0.741 |
| A reduced SMD after weighting indicates the weighting procedure distributed the covariates more equally across latent class membership (LCA model 3 – SAVA + social factors).  Combined weights include the overlap weights of latent class assignment, overlap weights of depression, inverse probability weights of lost-to-follow-up and latent class posterior probabilities. | | | |

| Supplementary Table 3. Summary statistics for comparing model fit between models with main effects only and interaction | | | | | |
| --- | --- | --- | --- | --- | --- |
|  | QIC | QICu | Quasi Lik | CIC | QUICC |
| LCA model 1: SAVA syndemic (homophobic bullying, general bullying, intimate partner violence, amphetamine use, binge drinking) | | | | | |
| Interaction - baseline | 1510.39 | 1399.95 | -693.97 | 61.22 | 1487.99 |
| Main effect - baseline | 1534.94 | 1469.15 | -730.58 | 36.89 | 1535.23 |
| Score test X2 | 6.6 | | | | |
| Score test P-value | 0.037 | | | | |
| Interaction - T4 | 850.10 | 834.02 | -411.01 | 14.04 | 827.70 |
| Main effect -T4 | 901.96 | 891.18 | -441.59 | 9.39 | 881.96 |
| Score test X2 | 9.91 | | | | |
| Score test P-value | 0.007 | | | | |
|  |  |  |  |  |  |
| LCA model 2: SAVA syndemic + internalized HIV stigma + low social support | | | | | |
| Interaction - baseline | 1535.58 | 1437.09 | -712.55 | 55.24 | 1513.18 |
| Main effect - baseline | 1619.93 | 1553.81 | -772.91 | 37.06 | 1599.93 |
| Score test X2 | 21 | | | | |
| Score test P-value | <0.001 | | | | |
| Interaction - T4 | 897.46 | 885.76 | -436.88 | 11.85 | 875.06 |
| Main effect -T4 | 993.68 | 959.09 | -475.55 | 21.29 | 973.68 |
| Score test X2 | 57 | | | | |
| Score test P-value | <0.001 | | | | |
|  |  | | | | |
| LCA model 3: SAVA syndemic + internalized HIV stigma + low social support + sex work + low income | | | | | |
| Interaction - baseline | 1636.43 | 1492.22 | -740.11 | 78.11 | 1614.03 |
| Main effect - baseline | 1692.21 | 1600.30 | -796.15 | 49.95 | 1672.21 |
| Score test X2 | 20.5 | | | | |
| Score test P-value | <0.001 | | | | |
| Interaction - T4 | 927.52 | 890.94 | -439.47 | 24.29 | 905.12 |
| Main effect -T4 | 974.27 | 947.12 | -469.56 | 17.57 | 954.27 |
| Score test X2 | 7.66 | | | | |
| Score test P-value | 0.022 | | | | |
| ICC: crude intraclass correlation.  ICC^b^ and 95%CI^b^: bootstrapped ICC and its 95% CI by bias-corrected accelerated method (BCa) with 3000 resampling trials. Bootstrapped ICC point estimate was derived as the mean between the bootstrapped 95% CI assuming a symmetric distribution of between latent class variance.  QIC: quasi-information criterion, model fit statistic for GEE models, robust to misspecified correlation matrix; a lower value indicate a better fit.  QICu: unadjusted QIC, similar to QIC but only when correlation matrix is correctly specified.  Quasi Lik: quasi likelihood used in calculation of QIC, a lower value indicates better fit.  CIC: corrected information criterion, number of independent correlation estimated; a higher number indicates more complex between latent class correlation.  QICC: corrected quasi information criterion, similar to QIC suitable for a small sample size.  Score test X2 and P-value: aka. the Lagrange Multiplier test to compare two nested models (interaction vs main effects only ). It is more suitable for GEE model comparison than a Wald test in a small sample situation and does not requires variance estimate, thus avoiding bias due to misspecification of GEE models. | | | | | |

| Supplementary Table 4. Additive effects (main effects) of clustering psychosocial adversities and depression on suicidality by marginal structural models | | | | | | |
| --- | --- | --- | --- | --- | --- | --- |
|  | Lifetime suicidality at baseline | | | Past 4 month suicidality at 12-month | | |
|  | *β_ow_* | 95% CI*_ow_* | *P_ow_* | *β_ow_* | 95% CI*_ow_* | *P_ow_* |
| LCA model 1: SAVA syndemic (homophobic bullying, general bullying, intimate partner violence, amphetamine use, binge drinking) | | | | | | |
| Class 1 (intercept) | 2.31 | 1.85 – 2.77 | **<0.001** | 2.76 | 2.35 – 3.17 | **<0.001** |
| Class 2 | 4.23 | 3.38 – 5.07 | **<0.001** | 1.17 | 0.36 – 1.99 | **0.005** |
| Class 3 | 2.85 | 2.03 – 3.67 | **<0.001** | 0.19 | -0.62 – 0.99 | 0.648 |
| Depression | 1.44 | 0.65 – 2.24 | **<0.001** | 1.53 | 0.62 – 2.44 | **0.001** |
|  |  |  |  |  |  |  |
| LCA model 2: SAVA syndemic + internalized HIV stigma + low social support | | | | | | |
| Class 1 (intercept) | 2.29 | 1.83 – 2.74 | **<0.001** | 2.92 | 2.57 – 3.27 | **<0.001** |
| Class 2 | 3.03 | 2.11 – 3.95 | **<0.001** | 0.32 | -0.17 – 0.80 | 0.205 |
| Class 3 | 3.74 | 2.99 – 4.48 | **<0.001** | 0.69 | 0.03 – 1.36 | **0.04** |
| Depression | 1.48 | 0.69 – 2.27 | **<0.001** | 1.22 | 0.54 – 1.89 | **<0.001** |
|  |  |  |  |  |  |  |
| LCA model 3: SAVA syndemic + internalized HIV stigma + low social support + sex work + low income | | | | | | |
| Class 1 (intercept) | 1.94 | 1.26 – 2.61 | **<0.001** | 3.12 | 2.52 – 3.72 | **<0.001** |
| Class 2 | 2.80 | 1.97 – 3.64 | **<0.001** | -0.06 | -0.75 – 0.62 | 0.858 |
| Class 3 | 4.79 | 3.44 – 6.15 | **<0.001** | 0.66 | -0.46 – 1.77 | 0.251 |
| Depression | 2.16 | 1.03 – 3.30 | **<0.001** | 1.61 | 0.62 – 2.61 | **0.001** |
| *β_ow_* , CI*_ow_, P_ow_* : coefficients, confidence interval and p-value from generalized estimating equation (GEE) estimating the average exposure [treatment] effects (ATE) from marginalized structural model by overlap weights (OW) with robust standard errors (sandwich) and clustering by latent class variable. | | | | | | |

| Supplementary Table 5. Comparing model estimates across weighting, trimming and modelling methods | | | | | | |
| --- | --- | --- | --- | --- | --- | --- |
|  | Lifetime suicidality at baseline | | | Past 4 month suicidality at T4 | | |
|  | *β* | 95% CI | *P* | *β* | 95% CI | *P* |
| **1.Overlap weights weighted GEE, untrimmed** | |  |  |  |  |  |
| Class 1 (intercept) | 3.03 | 3.00 – 3.06 | **<0.001** | 3.64 | 3.06 – 4.23 | **<0.001** |
| Class 2 | 1.53 | 0.59 – 2.47 | **0.001** | -0.47 | -1.08 – 0.14 | 0.131 |
| Class 3 | 3.08 | 1.85 – 4.31 | **<0.001** | -0.63 | -1.21 – -0.04 | **0.036** |
| Depression | 0.01 | -0.04 – 0.06 | 0.672 | -0.15 | -1.12 – 0.83 | 0.765 |
| Class 2 × depression | 2.50 | 1.12 – 3.88 | **<0.001** | 1.31 | -0.10 – 2.73 | 0.069 |
| Class 3 × depression | 3.61 | 1.12 – 6.09 | **0.004** | 3.85 | 1.23 – 6.46 | **0.004** |
|  |  |  |  |  |  |  |
| **2.Inverse probability weights weighted GEE, untrimmed** | | | |  |  |  |
| Class 1 (intercept) | 3.05 | 3.00 – 3.09 | **<0.001** | 3.56 | 3.03 – 4.09 | **<0.001** |
| Class 2 | 1.48 | 0.79 – 2.16 | **<0.001** | -0.26 | -0.86 – 0.34 | 0.398 |
| Class 3 | 3.22 | 1.97 – 4.48 | **<0.001** | -0.51 | -1.05 – 0.02 | 0.061 |
| Depression | 0.05 | -0.07 – 0.17 | 0.409 | -0.28 | -0.96 – 0.39 | 0.411 |
| Class 2 × depression | 2.55 | 1.46 – 3.65 | **<0.001** | 1.44 | 0.24 – 2.65 | **0.018** |
| Class 3 × depression | 3.54 | 1.04 – 6.04 | **0.005** | 4.64 | 2.60 – 6.68 | **<0.001** |
|  |  |  |  |  |  |  |
| **3. Inverse probability weights weighted GEE, trimmed** | | | |  |  |  |
| Class 1 (intercept) | 3.05 | 3.00 – 3.09 | **<0.001** | 3.57 | 3.04 – 4.11 | **<0.001** |
| Class 2 | 1.48 | 0.79 – 2.16 | **<0.001** | -0.27 | -0.88 – 0.33 | 0.372 |
| Class 3 | 3.21 | 1.96 – 4.46 | **<0.001** | -0.49 | -1.04 – 0.06 | 0.078 |
| Depression | 0.05 | -0.07 – 0.17 | 0.409 | -0.30 | -0.98 – 0.38 | 0.390 |
| Class 2 × depression | 2.55 | 1.46 – 3.65 | **<0.001** | 1.65 | 0.52 – 2.78 | **0.004** |
| Class 3 × depression | 3.55 | 1.06 – 6.05 | **0.005** | 4.48 | 2.41 – 6.55 | **<0.001** |
|  |  |  |  |  |  |  |
| **4. Unweighted GEE adjusted for confounding only** | | | |  |  |  |
| Class 1 (intercept) | 2.69 | 1.44 – 3.94 | **<0.001** | 3.37 | 2.26 – 4.49 | **<0.001** |
| Class 2 | 1.78 | 0.76 – 2.81 | **0.001** | -0.75 | -1.31 – -0.19 | **0.009** |
| Class 3 | 2.68 | 1.52 – 3.83 | **<0.001** | -1.32 | -2.18 – -0.46 | **0.003** |
| Depression | 0.11 | -0.14 – 0.35 | 0.496 | -0.12 | -0.74 – 0.50 | 0.714 |
| Class 2 × depression | 2.23 | 0.95 – 3.51 | **<0.001** | 0.96 | -0.01 – 1.93 | 0.053 |
| Class 3 × depression | 2.99 | 0.55 – 5.43 | **0.024** | 2.55 | 1.04 – 4.06 | **0.001** |
|  |  |  |  |  |  |  |
| **5. Unweighted GEE adjusted for baseline suicidality and confounding** | | | | |  |  |
| Class 1 (intercept) |  |  |  | 3.26 | 2.06 – 4.47 | **<0.001** |
| Class 2 |  |  |  | -0.74 | -1.30 – -0.17 | **0.011** |
| Class 3 |  |  |  | -1.33 | -2.20 – -0.46 | **0.003** |
| Depression |  |  |  | -0.13 | -0.75 – 0.49 | 0.677 |
| Class 2 × depression |  |  |  | 0.97 | -0.05 – 1.99 | 0.063 |
| Class 3 × depression |  |  |  | 2.62 | 1.13 – 4.12 | **0.001** |
|  |  |  |  |  |  |  |
| Unweighted generalized estimating equations adjusting for clustering by latent class variable and confounding effects of minimal sufficient variable sets from directed acyclic graphs (DAGs) including age, education, guardian’s education, income, time from HIV diagnosis and having a regular partner at past 6 months. | | | | | | |

| Supplementary Figure 1a. Directed acyclic graph (DAG) for LCA model 1- SAVA only. This DAG is viewable and editable online at <https://dagitty.net/dags.html?id=7R8AHZyc>  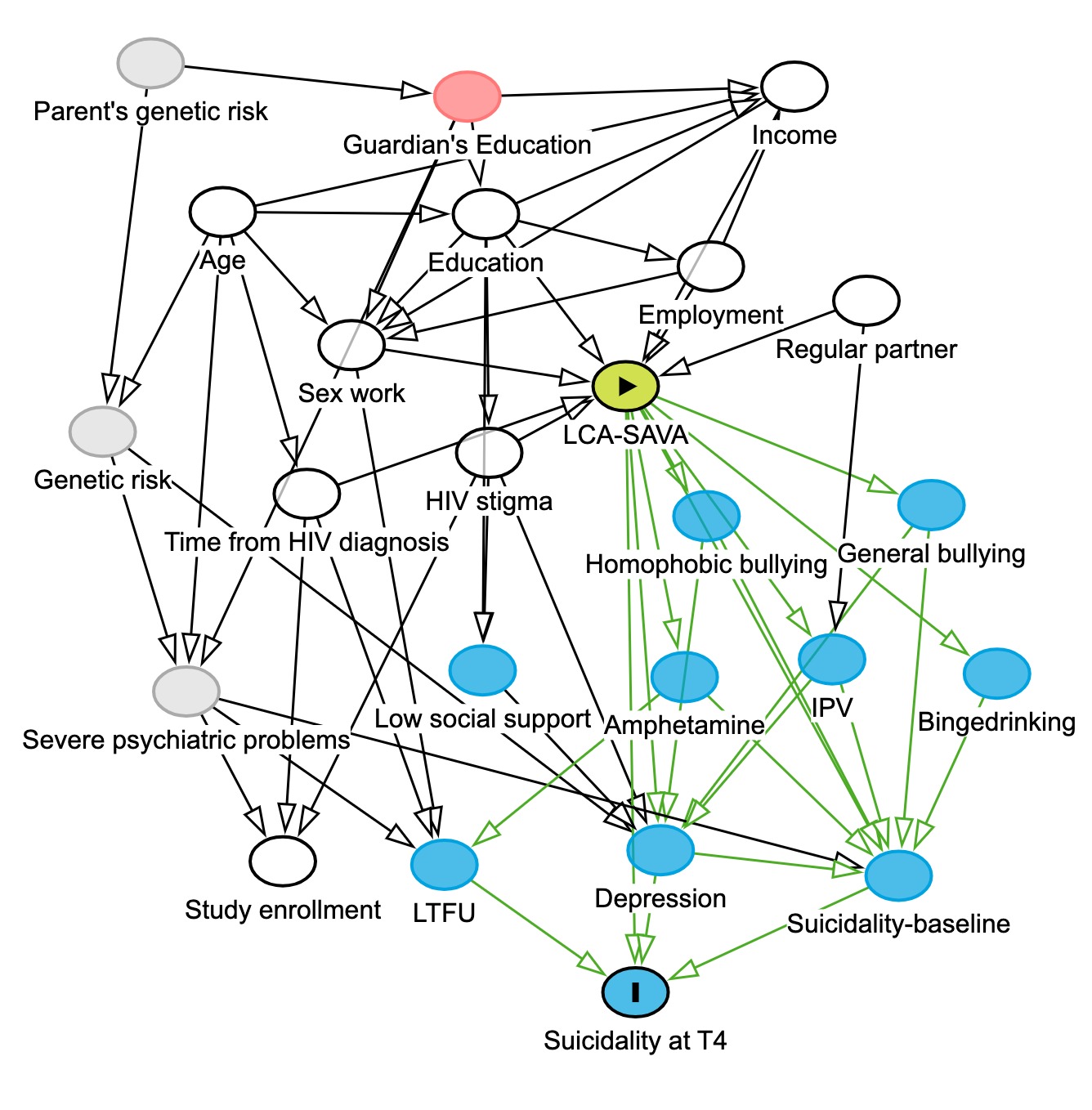  Supplementary Figure 1b. Boxplot showing the distribution of untrimmed overlap weight (OW) against extreme values for estimating the average exposure effect (ATE) of latent class model 1 (SAVA syndemic only) for suicidality at T4.  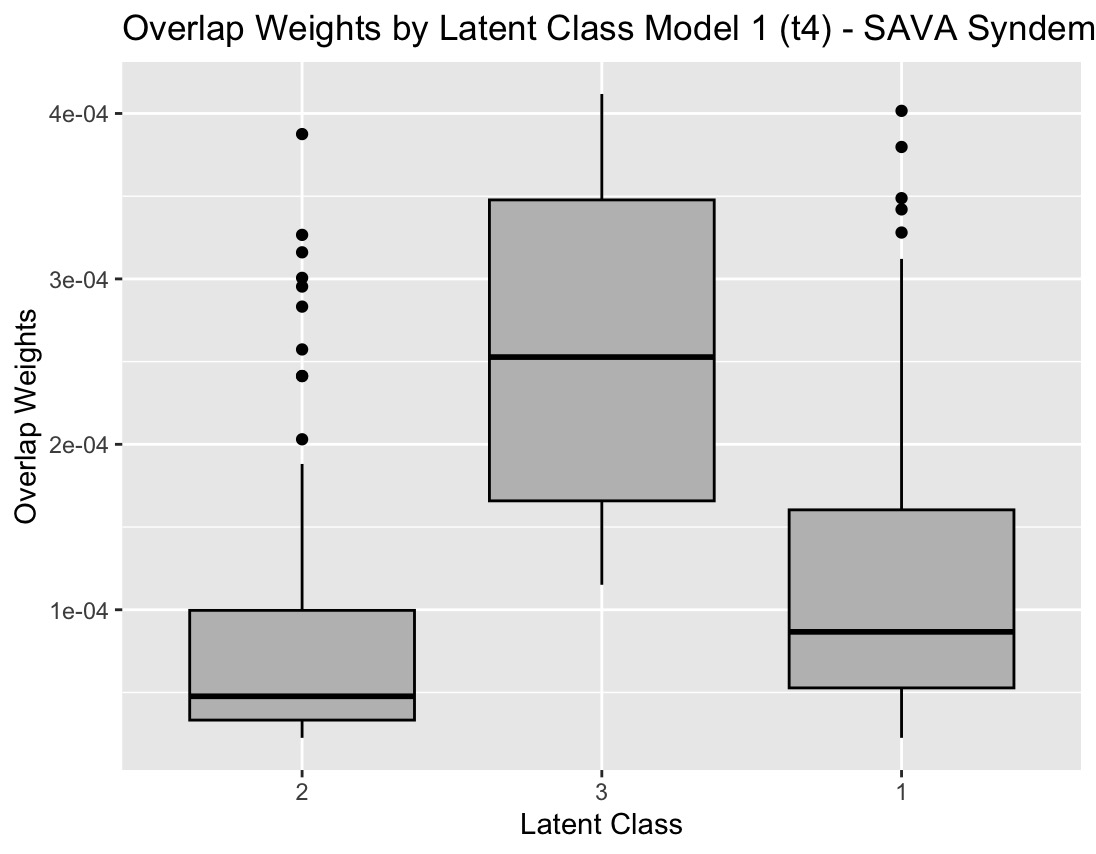  Supplementary Figure 1c. Density plot showing the distribution overlap of untrimmed overlap weight (OW) against skewedness, spread and multi-modal distribution for estimating average exposure effect (ATE) of latent class model 1 (SAVA syndemic only) for suicidality at T4.  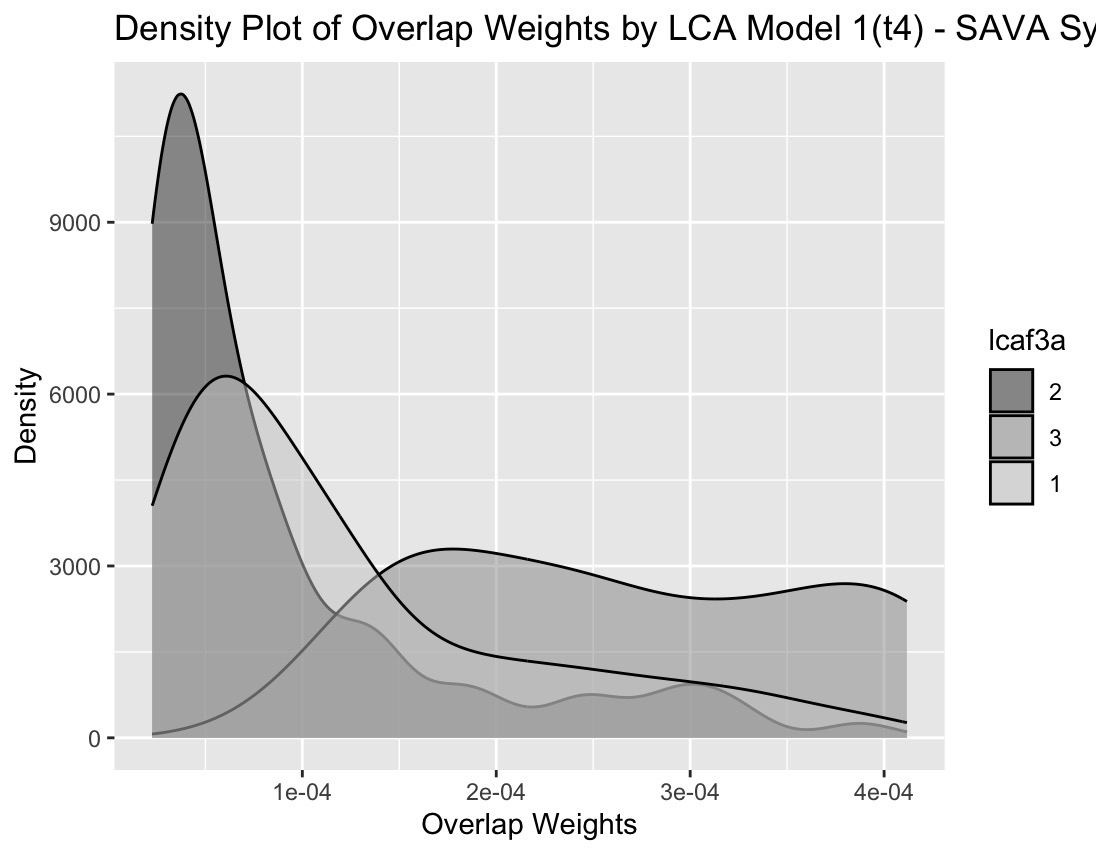 |
| --- |

| Supplementary Figure 2a. Directed acyclic graph (DAG) for LCA model 2- SAVA, HIV stigma and low social support. This DAG is viewable and editable online at <https://dagitty.net/mocbzwuKR>  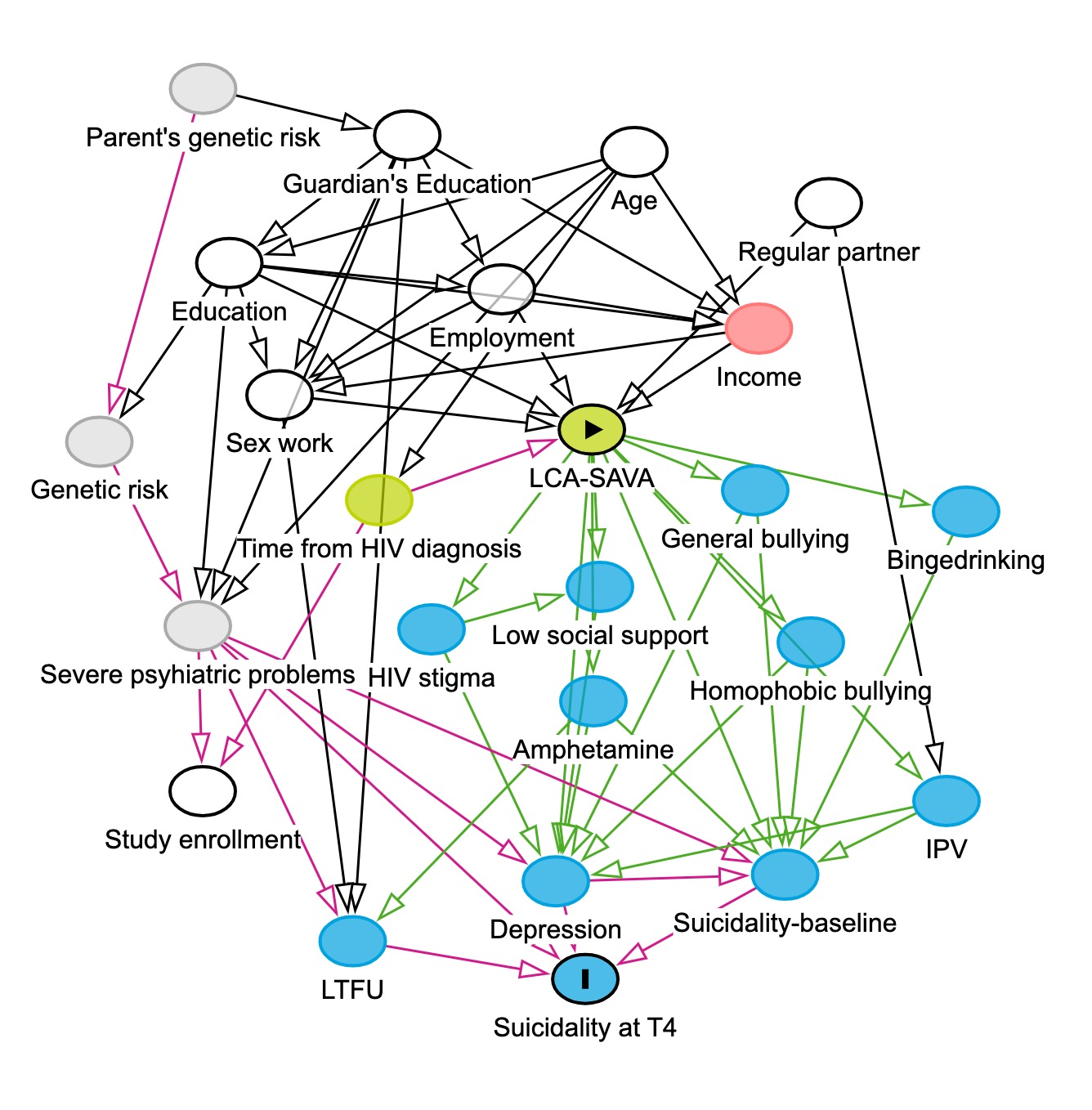  Supplementary Figure 2b. Boxplot showing the distribution of untrimmed overlap weight (OW) against extreme values for estimating the average exposure effect (ATE) of latent class model 2 (SAVA+) for suicidality at T4.  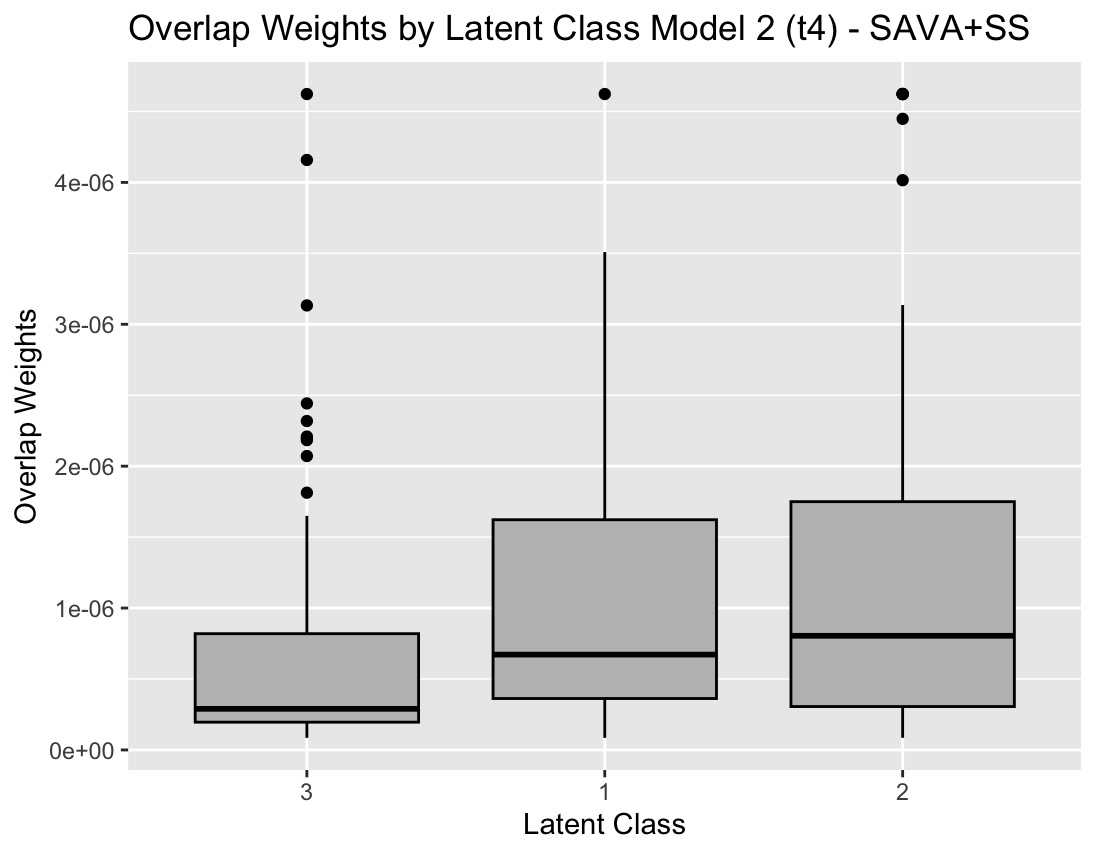  Supplementary Figure 2c. Density plot showing the distribution overlap of untrimmed overlap weight (OW) against skewedness, spread and multi-modal distribution for estimating average exposure effect (ATE) of latent class model 2 (SAVA+) for suicidality at T4.  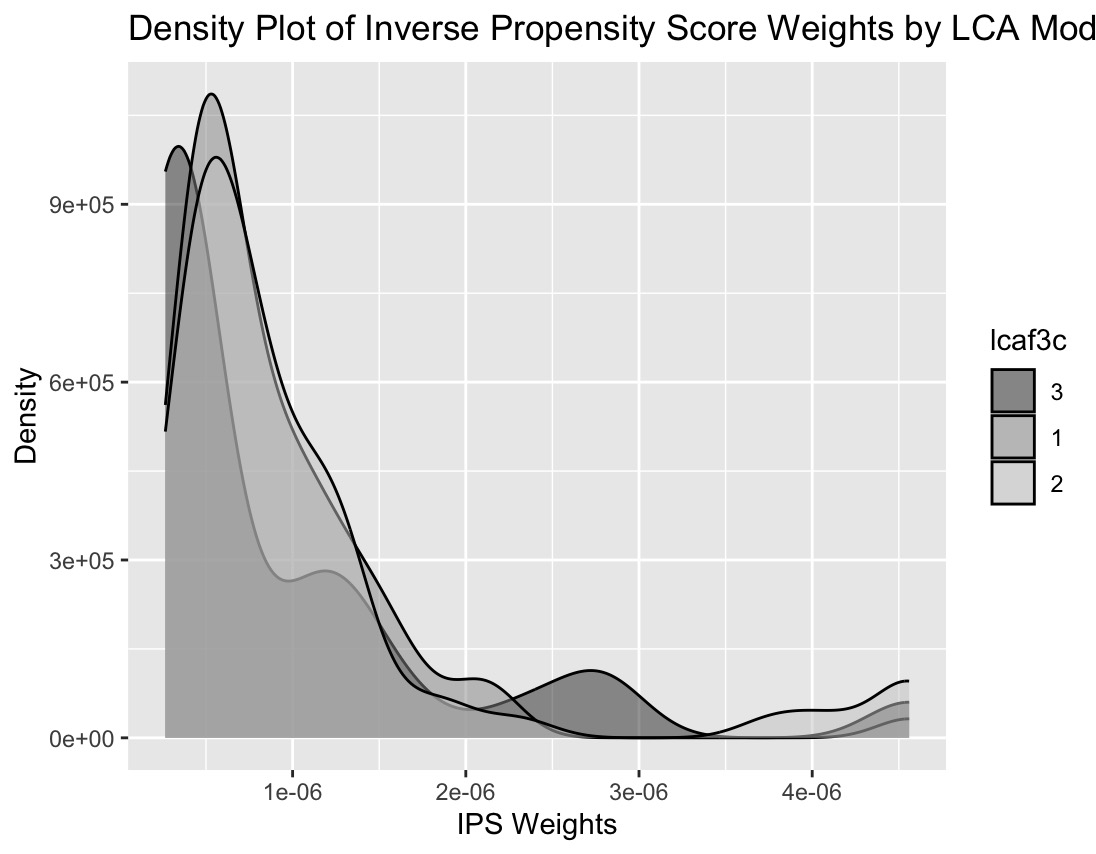 |
| --- |

| Supplementary Figure 3a. Directed acyclic graph (DAG) for LCA model 3- SAVA and social class. This DAG is viewable and editable online at https://dagitty.net/dags.html?id=3QW7F7uV  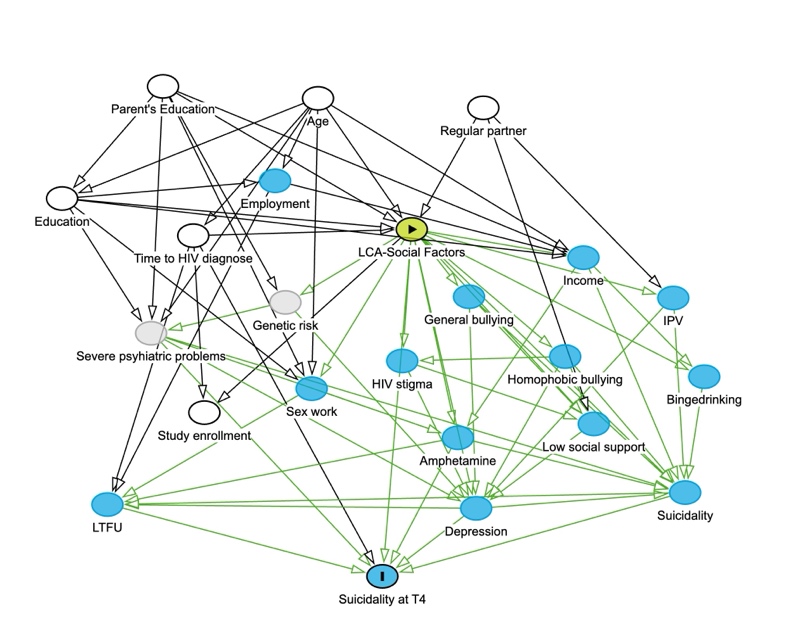  Supplementary Figure 3b. Boxplot showing the distribution of untrimmed overlap weight (OW) against extreme values for estimating the average exposure effect (ATE) of latent class model 3 (SAVA+social factors) for suicidality at T4.  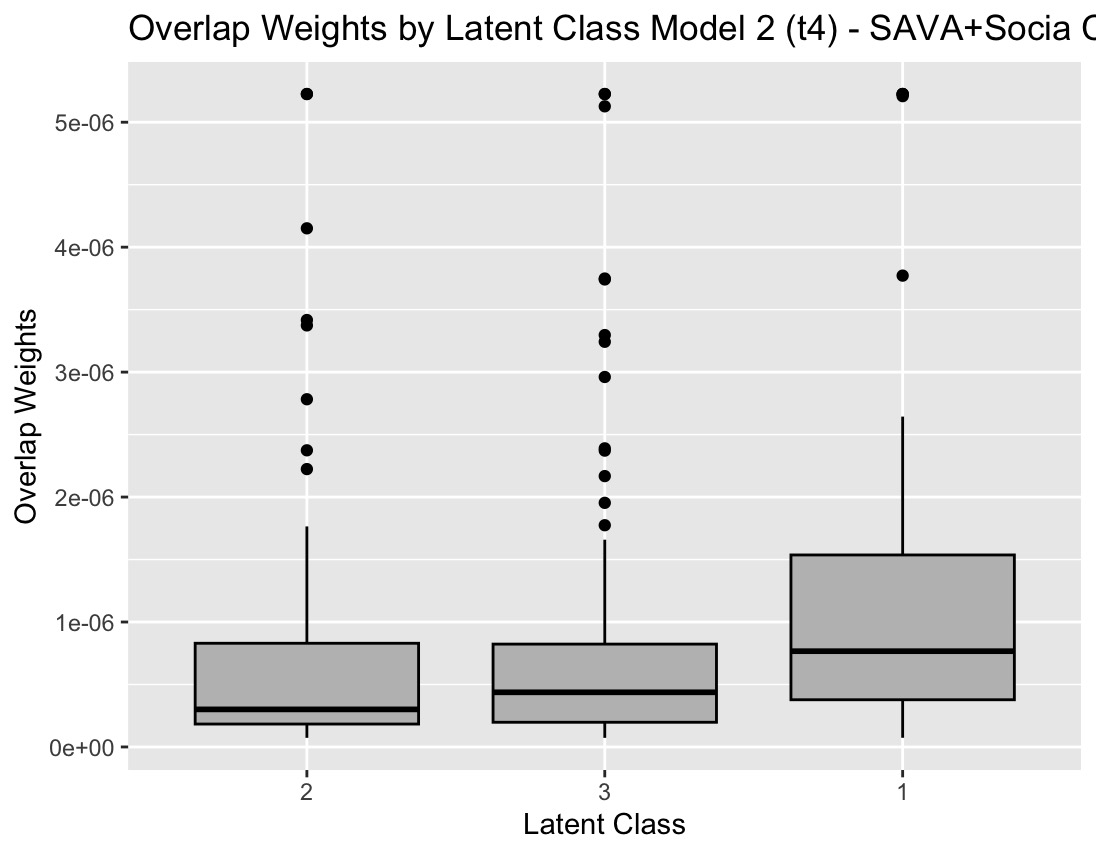  Supplementary Figure 3c. Density plot showing the distribution overlap of trimmed overlap weight (OW) against skewedness, spread and multi-modal distribution for estimating average exposure effect (ATE) of latent class model 3 (SAVA+ social class) for suicidality at T4.  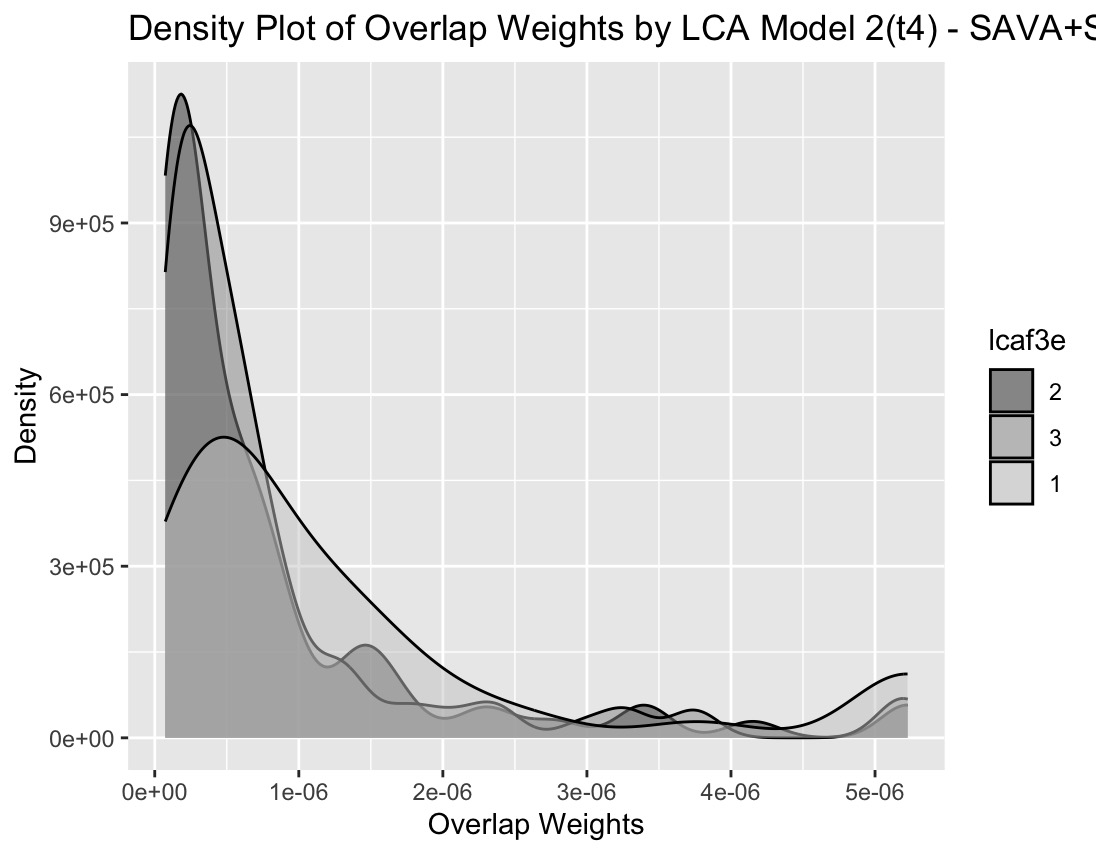 |
| --- |
